# Unable to walk or eat without assistance in pregnancy—Wilson’s disease in a resource-limited and conflict-affected setting: a case report

**DOI:** 10.1186/s13256-026-06273-8

**Published:** 2026-07-22

**Authors:** Taco Jan Prins, Nay Win Tun, Aung Myat Min, Sarah Alison Stokes, Rose McGready

**Affiliations:** 1 Department of Family Medicine, Faculty of Medicine, Chiang Mai University, Chiang Mai, Thailand; 2 Global Health and Chronic Conditions Research Group, Chiang Mai University, Chiang Mai, Thailand; 3 Amsterdam University Medical Centre, Department of Internal Medicine & Infectious Diseases, and Research Groups: APH, GH and AII&I, Amsterdam UMC, Amsterdam, The Netherlands; 4 Shoklo Malaria Research Unit, Mahidol–Oxford Tropical Medicine Research Unit, Faculty of Tropical Medicine, Mahidol University, Mae Sot, Thailand; 5 Centre for Tropical Medicine and Global Health, Nuffield Department of Medicine, University of Oxford, Oxford, UK

**Keywords:** Wilson’s disease, Pregnancy, Low resource setting, Conflict

## Abstract

**Background:**

Wilson’s disease, an autosomal recessive disorder, affects body copper metabolism. It can be fatal if left untreated as the copper accumulation ultimately leads to liver failure, brain damage, or both. In women discontinuation of menstruation, miscarriage and impaired conception can be symptoms of Wilson’s disease. Successful pregnancies in untreated patients with major symptoms are rare.

**Case presentation:**

A 30-year-old, gravidity 5, parity 4, Karen woman presented, after travelling for more than 5 h to a clinic on the Thailand–Myanmar border. Over the course of a year she progressively lost the ability to walk, sit or drink by herself. Kayser–Fleischer rings were present along with a 7-month pregnancy. Oral zinc was commenced based on a provisional diagnosis of Wilson’s disease, and later switched to D-penicillamine when it could be sourced. After birthing a healthy term female baby, mum required an assistant to secure and maintain attachment for breastfeeding. After 1 year of follow-up most of the symptoms had resolved and the baby was thriving.

**Conclusions:**

A thorough physical examination remains essential for neurological presentations in resource-limited and conflict-affected settings remains key to diagnosis and life-saving treatment, including for Wilson’s disease.

**Supplementary Information:**

The online version contains supplementary material available at 10.1186/s13256-026-06273-8.

## Background

Wilson’s disease, an autosomal recessive disorder with a prevalence of 1 in 30,000, affects body copper metabolism [1, 2]. A mutation in the ATP7B gene on chromosome 13 decreases or stops the transport of excess copper leading to accumulation in the liver and brain [1, 3]. Unfortunately it can be fatal if left untreated as the copper accumulation ultimately leads to liver failure, brain damage or both. Most of the people with Wilson’s disease will get symptoms typically diagnosed between the ages of 10–20 years, but exceptions are present: with reported diagnoses after the age of 70 years [4, 5]. Patients will present with only, or a mixture of, hepatic (jaundice, anorexia and emesis), neurologic (tremor, dystonia, dysarthria and or dysphagia) or psychiatric (depression, personality change and or anxiety) symptoms [4]. In women discontinuation of menstruation, miscarriage and impaired conception can be symptoms of Wilson’s disease [6]. During pregnancy the symptoms can increase [3, 6]. Successful pregnancies in untreated patients with major symptoms are rare [7].

## Case presentation

A 30-year-old, gravidity five, parity four, ethnic Karen woman presented after travelling for more than 5 h to reach a clinic in conflict-affected Myanmar. Supported by her husband they reported a 1-year history of increasing muscle cramps, then a gradual onset of a progressive bilateral tremor in the upper and lower limbs. At the time of presentation, the tremor was so severe that she was not able to walk, stand or sit by herself. She was carried to the bathroom, and food and water were provided by her mother, husband or children. She had a very quiet voice, appeared withdrawn, and had experienced weight loss during the current pregnancy (gestation confirmed by a late ultrasound). Her previous pregnancies were uncomplicated and she had delivered each of them at home. She did not consume alcohol. On further questioning her sister had developed a similar progressive tremor, and died at 20 years through a chest infection. Her family had already sought care at two other hospitals but were told nothing could be done.

**Fig. 1:**
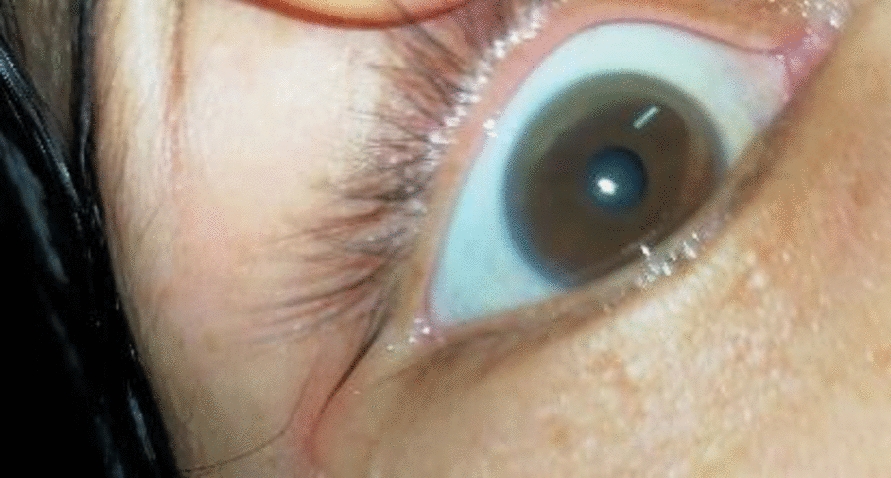
Kayser–Fleischer ring

On examination she had marked positional and intention tremor, (Supplementary Material 1: Video 1) which was bilateral but asymmetric (worse on the left). Her power was preserved throughout, there was no wasting, reflexes and sensation were normal, and coordination was limited largely by tremor. On examination of her eyes, Kayser–Fleischer rings were present bilaterally (Fig. 1). She had poverty of facial expression and hypophonia but no slurring of speech. Her eyesight was grossly normal, and while there was no nystagmus, she followed objects with delay. She had mild bilateral palmar erythema which may have related to her pregnancy. The cardio-respiratory examination was normal and there was no palpable hepatomegaly or signs of chronic liver disease.

Off-site investigations showed a mild anaemia, haemoglobin 9.7 g/dL (12–16), and slight increase of liver enzymes, Alanine Transferase 21 U/L (< 35), Aspartate Aminotransferase 37 U/L (< 35), Alkaline Phosphatase 137 U/L (30–120), Total Bilirubin 0.3 mg/dL (0.3–1.2), Direct Bilirubin 0.1 mg/dL (< 0.2), and Gamma-glutamyl Transferase 69 U/L (< 38). Other biochemistry values were normal. She was HIV, Hepatitis B and C, negative. The cluster of Kayser–Fleisher rings, bilateral and progressive tremor, and suspicious family history pointed to Wilson’s disease: serum ceruloplasmin was low at 0.091 g/L (normal range 0.2–0.6) and the 24-h urine copper was high at 2325 μG/day (normal < 35). Genetic testing, 6 months after first presentation, via a sample sent to Siriraj Hospital, Mahidol University, Bangkok Thailand, showed a homozygous pathogenic ATP7B variant (c.2692C > T(p.Gln898*) and the diagnosis of Wilson’s disease was confirmed.

The patient was initially treated with a low copper diet and oral zinc sulphate 30 mg orally, three times daily. Liver function tests repeated after 1 month, indicated rising Alkaline Phosphatase [240 U/L (30–120)] which may occur with increasing gestational age but also in zinc deficiency, and it was continued as the pregnancy was near term. D-penicillamine capsules [Samarth Life Sciences PVT (India)] 250 mg once daily, and Vitamin B6 (pyridoxine) 20 mg twice daily, were commenced. Skilled birth attendants at Shoklo Malaria Research Unit clinic supported a vaginal breech birth, without complications or oxytocic drugs at 39 + 1-week gestation, 2 weeks after commencement of the D-penicillamine. The female baby of 3010 g, had Apgar scores of 9 and 10 (at 5 and 10 min) and a normal newborn surface examination, including cardio-respiratory auscultation, and absence of Kayser–Fleischer rings. The mother wanted to breastfeed and required support as she could not physically hold the child. She agreed to a 5-year implant as a post-partum contraceptive. At 2-month post-partum her symptoms were improved and she was able to walk without support. (Supplementary Material 2: Video 2) Currently, after 1 year and 5 months, the symptoms have significantly improved on daily D-penicillamine (adjusted over time; maximum daily dose 875 mg), pyridoxine and zinc, and her baby remains healthy. (Supplementary Material 3: Video 3). All the live children (*n* = 4; her second born child was an early neonatal death) have undergone testing for serum ceruloplasmin and 24-h urine copper excretion, which indicate they are unlikely to have Wilson’s disease; however, they remain under follow-up.

The biggest barrier to this patient receiving appropriate care was reaching a clinic within conflict-affected Myanmar that did a complete neurological examination. In person follow-up has been adapted to be less frequent than recommended for D-penicillamine with periods of up to 6 months between visits; due to difficulty of travel within a conflict zone, and exceptional travel costs.

## Discussion

This report of Wilson’s disease with severe neurologic symptoms with a diagnosis in pregnancy is unique. Most women can only become pregnant when they are asymptomatic or have mild symptoms [6, 7]. Despite experiencing symptoms for a year and being completely reliant on others for survival due to severe neurological issues, this patient showed minimal liver involvement, as indicated by only slightly elevated liver function tests at 30 weeks into her pregnancy. When the liver is damaged by copper accumulation, it cannot inactivate oestrogen, which could lead to negative feedback to the hypothalamus–pituitary–ovarian axis [6, 8]. Another reason for difficulties with conceiving in patients with symptoms could be a result of the deposit of copper in the endometrium which has a contraceptive effect [8, 9]. In treated Wilson’s disease there have been previous reports of pregnancies with good outcomes [3, 8, 10–13].

Management of non-communicable and genetically acquired disease during pregnancy is challenging with patients in resource-limited and conflict-affected settings facing additional financial and access barriers. For Wilson’s disease, zinc therapy is less expensive than D-penicillamine and can be used as monotherapy, unless there is decreased copper excretion due to liver involvement [14, 15]. D-penicillamine is known to be teratogenic in animals and birth defects have been reported with the use of D-penicillamine [6, 11]. In this case symptoms, drug availability, gestational age and patient choice, i.e., the desire to breastfeed, were factored into medication choices. Breastfeeding is safe with the use of Zinc and/or D-penicillamine [16]. The severity of the symptoms and hepatic involvement were reasons to commence D-penicillamine and the late gestation of presentation negated teratogenic effects. The treatment plan for this patient is to shift to lifelong Zinc monotherapy. In this case there was no decompensated liver cirrhosis, coagulation disorder, or oesophageal varices, and no obstetric indication for caesarean section [6, 8].

Our patient presented at a late stage after leaving other health facilities without a diagnosis: 1 year after the onset of her symptoms, and already reliant on her family for care due to advanced neurological disease. Barriers to accessing functional healthcare, lack of financial resources, and transportation difficulties, including distance and concerns over security, contributed to the very late presentation; and as discussed by the family it is also the reason that her sister never received a diagnosis. The country where the patient lives, Myanmar, has witnessed an increasing number of people who are in need of humanitarian aid since the military coup in 2021: estimated at 18.6 million people on a population of 56.6 million people in 2023 [17]. Health service disparities existed prior to the military takeover in Myanmar but these have been compounded by the successive problems of COVID-19 pandemic (March 2020 full lockdown) and the most recent coup d’etat (February 2021) [17, 18]. Fortunately this patient managed to reach care where she was diagnosed and started on effective treatment and provided free perinatal care by skilled birth attendants who spoke her language. She is now able to take care of herself, her baby and the rest of her family.

## Conclusion

Severe neurological presentations in reproductive age and pregnant females are not all hopeless. In resource-limited and conflict-affected environments, a thorough physical examination is nevertheless essential for diagnosis and consequently appropriate therapy. In this case a Wilson’s Disease diagnosis and D-Penicillamine, Zinc and Pyridoxine treatment; saved two lives.

## Supplementary Information

Additional file 1.

Additional file 2.

Additional file 3.

## Data Availability

All data underlying the results are available as part of the article and no additional source data are required.
